# Müller and macrophage-like cell interactions in an organotypic culture of porcine neuroretina

**Published:** 2008-11-28

**Authors:** Ivan Fernandez-Bueno, Jose Carlos Pastor, Manuel Jose Gayoso, Ignacio Alcalde, Maria Teresa Garcia

**Affiliations:** 1 University of Valladolid, Instituto Universitario de Oftalmobiologia Aplicada (IOBA), Campus Miguel Delibes, Valladolid, Spain; 2 University of Valladolid, Faculty of Medicine, Celullar Biology, Histology and Pharmacology Department, Ramon y Cajal nº 7, Valladolid, Spain

## Abstract

Purpose: To analyze the in vitro Müller cell modifications in an organotypic culture of porcine neuroretina in response to the addition of a blood-derived mononuclear fraction (MNF; monocytes and lymphocytes) as a source of macrophages.

Methods: Control and MNF-stimulated neuroretinal explants were examined at 3, 6, and 9 days of culture. Specimens were processed for epoxy-resin embedding and cryosectioning. Light and immunofluorescence microscopy were performed, using toluidine blue staining and antibodies against glial fibrillary acidic protein (GFAP), as a reactive gliosis marker, and cellular retinaldehyde-binding protein (CRALBP), as a Müller cell marker.

Results: Compared to controls, explants cocultured with MNF displayed increased cellular disorganization and larger tissue invasion of the subretinal space at 9 days of culture. Immunostaining of the MNF-treated explants revealed evidence of more reactive gliosis and greater number of GFAP-immunoreactive Müller cells that had increased width and processes extending into the subretinal space and forming a multilayer tissue. Astrocytes also responded to the MNF addition, producing extensions that invaded the neuroretinal outer layers.

Conclusions: Addition of MNF stimulates modifications of Müller cells, producing a wider intraretinal reactive gliosis and tissue proliferation at the subretinal space (outer layers of the retina). These findings emphasize the role of macrophage-like cells in the production of changes in retinal structure observed after retinal detachment in humans.

## Introduction

In the normal retina, Müller cells surround neurons in the retinal tissue [1] and have nuclei located at the inner nuclear layer (INL). The cytoplasm of Müller cells extends from the inner limiting membrane (ILM) to the outer limiting membrane (OLM). After retinal detachment (RD), multiple modifications take place at the cellular level of the neuroretina, including significant changes in Müller cells. The nuclei become translocated to the external layers, and cell bodies become hypertrophic and hyperplastic. Their processes extend into the subretinal space, forming subretinal membranes and initiating a reactive gliosis [2-6]. All these findings have been described in experimental models and confirmed in human retinal samples obtained during retinectomies from proliferative vitreoretinopathy (PVR) cases [6-10].

Our group has previously reported a strong correlation between the development of PVR and the presence of macrophage-like cells in vitreous samples [11]. These cells, which are CD68 positive and cytokeratin negative, are also present in human retinal tissue obtained from PVR retinectomy specimens [9]. They probably derive from blood monocytes and are absent in normal human retina [12]. They infiltrate the retina and probably activate Müller glial cells, possibly via tumor necrosis factor alpha (TNFα), as proposed in an experimental model of choroidal neovascularization [13]. These and other findings [14-18] emphasize the important role of macrophages in PVR development. The purpose of this work is to analyze the in vitro response of Müller cells to a mononuclear blood fraction containing monocytes and lymphocytes as a source of macrophages.

## Methods

### Neuroretina explant preparation and organotypic culture

Nineteen eyes from domestic pigs, age 6–8 months old, were obtained from the local slaughterhouse and immersed in ice-cold transport medium composed of Dulbecco’s Modified Eagle Medium (DMEM) CO_2_-independent medium without L-glutamine. This medium was supplemented with 1% antibiotic–antimycotic mixture containing penicillin, streptomycin, and amphotericin B (Gibco, Paisley, UK). Eyes were transported on ice to the laboratory where, under aseptic conditions, each eyeball was immersed in 70% ethanol and washed in transport medium. With blunt scissors, all extraocular tissues were removed. Then the sclera was punctured with a 22 gauge needle at the ora serrata and bisected with corneal scissors, dividing the ocular globe into anterior and posterior eyecups. The vitreous was removed, and the posterior eyecup was placed into a dish with clean transport medium. A paintbrush was used to mechanically detach the neuroretina from the retinal pigmented epithelium (RPE), and the optic nerve was cut with Westcott scissors. The neuroretina was unrolled and cut into 5×5 mm explants, in such a way as to avoid visible blood vessels. Explants were transferred to Transwell^®^ culture dishes (Corning Inc., Corning, NY), containing 1.6 ml culture medium composed of Neurobasal A medium supplemented with 10% fetal bovine serum, 2% B-27 (Gibco), 1% L-glutamine (Sigma-Aldrich, St. Louis, MO), and 1% antibiotic–antimycotic mixture. Explants were cultured at 37 °C with 5% CO_2_ in a humidified atmosphere. The culture medium level was maintained in contact with the support membrane beneath the explant and changed with freshly prepared, warmed medium on days 1, 3, 5, and 7. Specimens were collected at culture days 3, 6, and 9. Freshly detached neuroretinas were also obtained for normal morphologic evaluation.

### Mononuclear fraction extraction and addition

Blood from the marginal vein of each animal was collected to prepare the mononuclear fraction (MNF) from which macrophages were derived. The blood was transported to the laboratory in heparinized collection tubes (Heparina Leo 1%; Byk Leo, Madrid, Spain), and MNF extraction begun 30 min after collection. Next, 6 ml heparinized blood was diluted 1:1 in 0.1 M filtered phosphate buffered saline (PBS, pH 7.4) and mixed by inversion. Histopaque^®^-1077 (Sigma-Aldrich) was used to separate the MNF. Histopaque^®^ was deposited in a centrifuge tube, and two volumes of blood-PBS mixture were added. Tubes were centrifuged at 400x g for 30 min, obtaining an opaque interface containing the MNF. The plasma was removed and then the MNF was taken, leaving the erythrocytes and polymorphonuclear leukocytes in the tube. The MNF was placed into a clean centrifuge tube and washed in 10 ml PBS by centrifugation at 250x g for 10 min, producing a pellet at the bottom of the tube. The supernatant was removed and 200 μl of retinal culture medium was directly added over the pellet to reconstitute it. The cell count, made with a Neubauer counting chamber, was about 15×10^6^ cells/ml. Cell viability, determined by the trypan blue dye exclusion method [19], was always greater than 90%.

At day 0, 20 ml of the MNF cell suspension were added over each explant. To ensure that mononuclear cells were deposited and remained on the retina during the culture, we placed a plastic cylinder, which had an inner diameter of 4 mm, on each retina, and deposited the MNF into it. To verify the presence of monocytes in the MNF, we put a drop on a glass slide, which was then smeared and air-dryed. The slide was stained by the Giemsa method [20] and examined under light microscopy. To verify the survival of macrophage-like cells, we cultured the remaining MNF in 3 ml of retinal culture medium. After 2 days of culture, the medium was removed, and the cells were stained with the Giemsa method and examined by light microscopy.

### Light and immunofluorescence microscopy

Specimens were fixed for a maximum of 6 h at 4 °C in 4% filtered paraformaldehyde (Panreac Química S.A., Barcelona, Spain) in 0.1 M PBS (pH 7.4) and cryoprotected in 30% saccharose (Panreac Química S.A.) at 4 °C for 24 h. Samples were frozen embedded in Tissue-Tek^®^ O.C.T. ^™^ Compound (Sakura Finetek Europe B.V., Zoeterwoude, The Netherlands). Next, 12 μm sections were cut with a cryostat (Leica, Nussloch, Germany) and placed on commercially treated slides (Fisher-Biotech, Pittsburgh, PA).

A double immunostaining protocol was performed. The first stain was for glial fibrillary acidic protein (GFAP), which constitutes the intermediate filaments (IF) that are prominent in Müller cells and astrocytes exhibiting reactive gliosis [21-23]. The second was for cellular retinaldehyde-binding protein (CRALBP), a retinoid-binding protein implicated in vitamin A metabolism [24]. CRALBP expression is always found in Müller cells and in astrocytes only during the first two postnatal weeks [24,25]. Colabeling of cells with both antibodies allowed differentiation of Müller cells from astrocytes [25,26]. Primary antibodies against undiluted, polyclonal rabbit anticow GFAP (DakoCytomation Inc., Carpinteria, CA) incubated for 30 min at room temperature and 1:1,000 CRALBP (mouse monoclonal antibody [B2]; Abcam plc., Cambridge, UK) incubated overnight at 4 °C were used. Following primary antibody incubation, sections were incubated with secondary antibodies composed of 1:200 dilution each of Alexa Fluor^®^594 goat antirabbit and Alexa Fluor^®^488 goat anti-mouse (Molecular Probes, Eugene, CA) respectively for 2 h. Cellular nuclei were stained with 10 μg/ml 4’,6-diamino-2-phenilindole dihydrochloride (DAPI; Molecular Probes) for 10 min. The slides were then coverslipped with 1:1 PBS-glycerol.

For epoxy-resin embedding, specimens were fixed as described in the previous section and dehydrated in graded alcohols. Tissue infiltration was made in graded concentrations of propylene oxide–epoxy resin-Araldite (TAAB, Berks, UK), and finally resin was polymerized at 60 °C for 24 h. Next, 1 μm sections  were cut with an ultramicrotome (LKB, Bromma, Sweden) and mounted on slides treated with (3-aminopropyl) triethoxy-silane (Sigma-Aldrich). For light microscope evaluation, sections were stained with toluidine blue and coverslipped with Entellan^®^ (Merck, Darmstadt, Germany). For semithin immunohistological staining, a published method [7] was modified. Resin-embedded sections were washed in propylene oxide, and epoxy resin was removed in a sodium ethoxide solution. Subsequently, sections were dehydrated in graded acetones. Immunostaining was performed as described in the previous section using antibody against GFAP and DAPI dye.

Sections of porcine optic nerve were used as positive controls for anti-GFAP and anti-CRALBP antibodies. Negative controls included substitution of the primary antibody with PBS and secondary antibody omission. Light and immunofluorescence studies were performed with an Axiophot microscope (Zeiss, Oberkochen, Germany) equipped for epifluorescence. Images were captured with a Spot digital camera SP402–230 (Diagnostic Instruments, Sterling Heights, MI) and processed with the appropriate software (Spot Advanced Version 3.5.9. for Windows; Diagnostic Instruments). Fluorescence was also detected with a confocal microscope imaging system (Leica TCS SP2; Leica, Wetzlar, Germany) equipped with an Ar-Kr laser. One Airy unit was used, giving an optical slice thickness less than 0.9 μm. TIFF images were enhanced using Adobe Photoshop software (Version 10.0.1 for Macintosh).

## Results

### Control porcine neuroretina in organotypic culture

#### Macroscopic morphology

A total of 75 control explants were cultured for different periods (Table 1). Eight samples lost their transparency in the first days of culture and were discarded. The other explants remained transparent and became thinner during the course of the culture. This reduction of the retinal thickness was more accentuated in the last days, making visible the normal retinal blood vessels at the superficial layers of the explants.

**Table 1:** Distribution of cultured explants.

**Culture days**	**Control explants**		**Explants+MNF**	
	**Cultured**	**Lost**	**Cultured**	**Lost**
3	25	1	13	0
6	25	3	13	1
9	25	4	13	1
	75	8	39	2
Total: 114 explants				

Control and MNF-stimulated neuroretinal explants were cultured during 3, 6 and 9 days. A total of 114 explants were used initially. Lost explants were disposed of in the experiment due to an opacification in the first days of culture. Mononuclear fraction (MNF) was added to explants in culture at day 0.

#### Light microscopy

**Figure 1:**
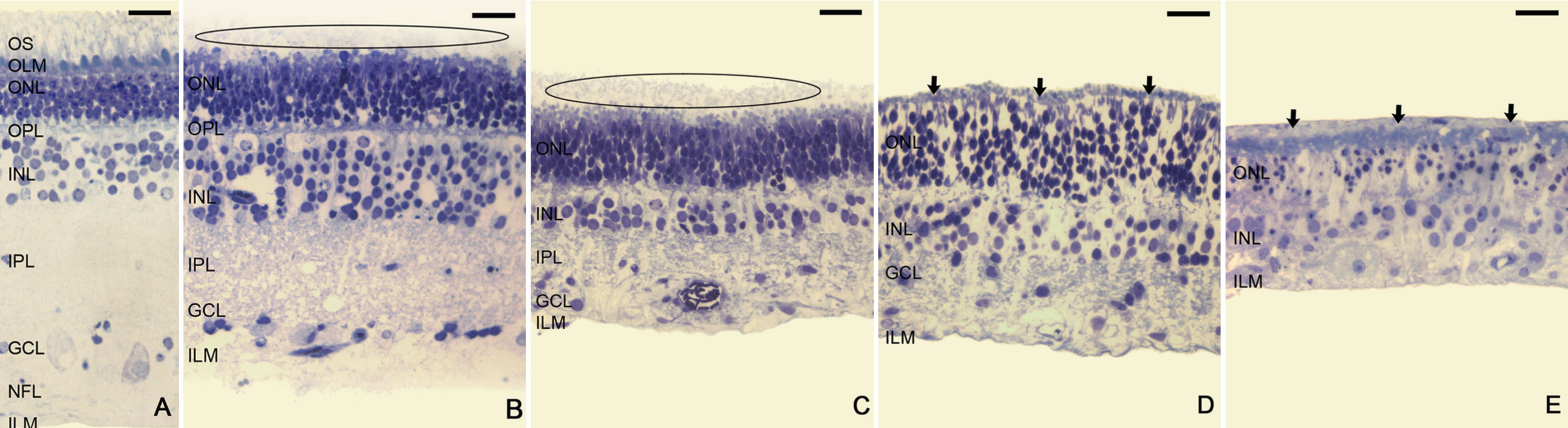
Toluidine blue staining of semithin sections from control explants. Neuroretinal morphology was preserved after experimental retinal detachment (**A**). At day 3 of culture (**B**), photoreceptor outer segments (OS) were truncated and disrupted and the neuroretina began to degenerate and become thinner. Vacuolization of the ganglion cell layer (GCL) and inner nuclear layer (INL) was apparent. The outer nuclear layer (ONL) showed 6–7 rows of photoreceptor nuclei as in post-detached specimens, but at this time point they were loosely distributed. Fragmented OS were present in the subretinal space (ellipsoid area). After 6 days of culture (**C**), the number of rows of nuclei was reduced in the INL, but remained constant in the ONL. The neuroretinal thickness continued to decrease, mainly due to narrowing of the plexiform layers. Fragmented OS remained present in the subretinal space (ellipsoid area). After 9 days in culture, explants that maintained the retinal architecture (**D**) revealed a lower packing density of cells, and there was a marked reduction in the number of nuclei in the INL. The plexiform layers almost disappeared and a tissue layer was present outside the outer limiting membrane (OLM; arrows). In explants that showed cellular disorganization (**E**), nuclei were randomly arranged, appearing outside the OLM and comprised a new multinuclear tissue layer in the subretinal space (arrows). Comparing (**A-E**) images, the retinal thinning during the culture is apparent. On days 3, 6, and 9, the ONL looked progressively thicker (**B-D**). Scale bar equals 20 µm. Abbreviations: inner nuclear layer (INL); nerve fiber layer (NFL).

The overall architecture of the neuroretina, including the delicate structures of photoreceptor inner segments (IS) and outer segments (OS), was well preserved immediately after mechanical detachment from the underlying RPE (Figure 1A). At day 3 of culture (Figure 1B), the neuroretina was thinner, and a vacuolization of the ganglion cell layer (GCL) and INL was apparent. OS were truncated and disrupted. In contrast, the IS appeared quite similar to those in the postdetachment specimens, in which the outer nuclear layer (ONL) had 6–7 rows of photoreceptor nuclei that were loosely distributed. The rest of the retinal architecture appeared fairly normal.

After 6 days of culture (Figure 1C), a considerable diminution of the neuroretinal thickness was apparent, mainly due to the thinning of the plexiform layers. The number of rows of nuclei was remarkably reduced in INL but remained constant in ONL. At culture days 3 and 6, fragmented OS were present in the subretinal space (Figures 1B,C, ellipsoid area). On days 3, 6, and 9, the ONL looked progressively thicker (Figures 1B-D).

After 9 days in culture, 9 of the surviving explants maintained similar retinal architecture as specimens incubated for 6 days (Figure 1D). The cells were less densely packed, and the INL showed a marked reduction in the number of nuclei. The plexiform layers had almost disappeared and a tissue layer was present outside the OLM. In addition, 12 of the other surviving explants displayed cellular disorganization (Figure 1E), losing the typical retinal structure. Nuclei were randomly arranged across the neuroretina and some were located outside the OLM, comprising a new multinuclear tissue layer oriented parallel to the photoreceptor surface.

#### Immunofluorescence microscopy

**Figure 2:**
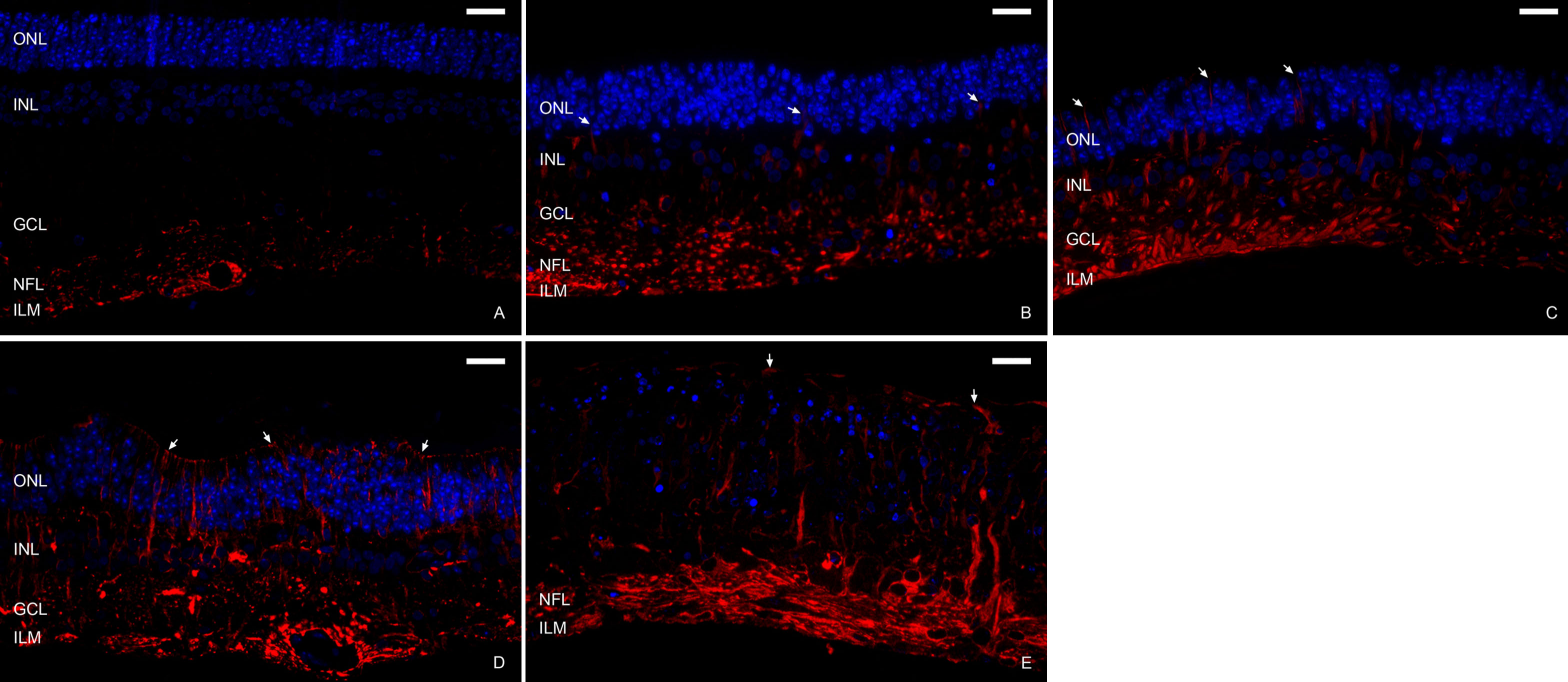
Immunofluorescence staining of semithin sections from control explants. Antibodies against glial fibrillary acidic protein (GFAP; red) were used to identify glial IF. DAPI dye (blue) was used to label nuclei. In newly detached samples (**A**), GFAP was present in the end feet of Müller cells (inner limiting membrane, ILM) and in astrocytes (nerve fiber layer, NFL). The outer nuclear layer (ONL), inner nuclear layer (INL), and ganglion cell layer (GCL) were identified with DAPI dye. At 3 days of culture (**B**), GFAP was detectable throughout the Müller cell cytoplasm, from the ILM to the ONL (arrows). After 6 days of culture (**C**), the Müller cells were wider and their GFAP+ processes reached the outer limiting membrane (OLM;  note arrows). After 9 days in culture, in explants that maintained the retinal structure (**D**), labeled processes extended beyond the OLM and began to create a continuous layer in the subretinal space (arrows). In samples that lost the characteristic retinal organization (**E**), nuclei of surviving cells and GFAP+ extensions were randomly distributed, appearing over the OLM (arrows). Scale bar equals 20 µm.

In semithin sections processed for fluorescence microscopy, the ONL, INL, and GCL were identified by DAPI staining. In freshly detached specimens (Figure 2A), GFAP+ IF were only detectable in the end feet of Müller cells at the ILM, and in astrocytes located in the nerve fiber layer (NFL).

At 3 days of culture (Figure 2B), GFAP reactivity increased compared to that in newly detached neuroretinas. Anti-GFAP and DAPI colabeling showed that during the culture period, the immunoreactive labeling extended throughout the length of the Müller cells from the ILM to the ONL. After 6 days of culture (Figure 2C), the cytoplasm of Müller cells was wider and the GFAP+ processes reached the OLM and extended beyond this structure.

After 9 days in culture, GFAP immunoreactivity reached its maximum in this experiment. In explants that maintained the retinal structure (Figure 2D), labeled extensions crossed the OLM and began to create a continuous layer parallel to that membrane. At this time some samples had lost the characteristic retinal organization (Figure 2E). In these explants, nuclei of surviving cells and GFAP+ extensions were randomly distributed. These also appeared outside the OLM.

**Figure 3:**
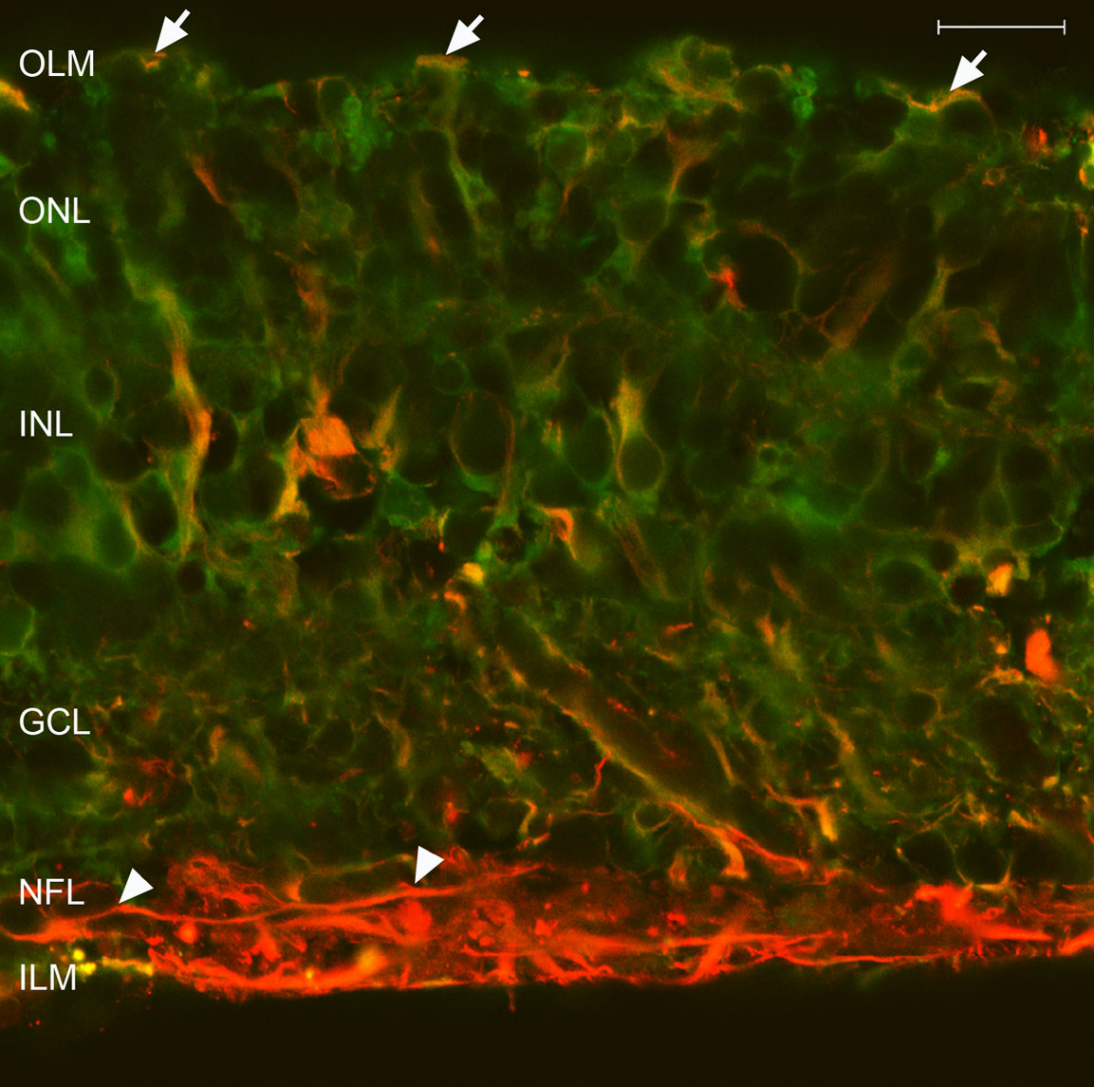
Immunofluorescence staining of cryosections from 6 day control explants. As seen in confocal images of cryostat sections, antibodies against glial fibrillary acidic protein (GFAP; red) identified glial intermediate filaments (IF), and antibodies against cellular retinaldehyde-binding protein (CRALBP; green) identified a retinoid-binding protein present in Müller cells. CRALBP labeling was more concentrated at the neuroretinal outer layers. Colocalization of both antibodies (yellow) marked Müller cells GFAP+. In control explants, only some Müller cells showed GFAP expression in the cytoplasm that reached the outer limiting membrane (OLM; arrows). Astrocyte cell bodies and GFAP+ extensions were located along the nerve fiber layer (NFL; arrowheads). Scale bar equals 20 µm. Abbreviations: ganglion cell layer (GCL); inner limiting membrane (ILM); inner nuclear layer (INL); outer nuclear layer (ONL).

To corroborate Müller cell identification by anti-GFAP staining and to allow differentiation of these cells from astrocytes, we colabeled cryosections of explants on day 6 of culture with both anti-GFAP and anti-CRALBP antibodies (Figure 3). Confocal images revealed that anti-CRALBP labeling was localized to the cytoplasm and extensions of the Müller cells, and it was more concentrated at the neuroretinal outer layers. A small number of these cells were GFAP+ and reached the OLM. Cell bodies and GFAP+ extensions of astrocytes were localized within the NFL.

### Variations in the porcine neuroretina organotypic culture after MNF addition

#### Macroscopic morphology

A total of 39 explants were cultured in the presence of the MNF for different periods (Table 1). Two of the samples lost transparency in the first days of culture and were discarded. During the culture, viable explants showed the same aforedescribed evolution in controls.

#### Light microscopy

Histological smears of MNF stained with the Giemsa method showed abundant monocytes and lymphocytes. After 2 days in culture with the retinal medium, high numbers of macrophage-like cells appeared in the MNF culture.

**Figure 4:**
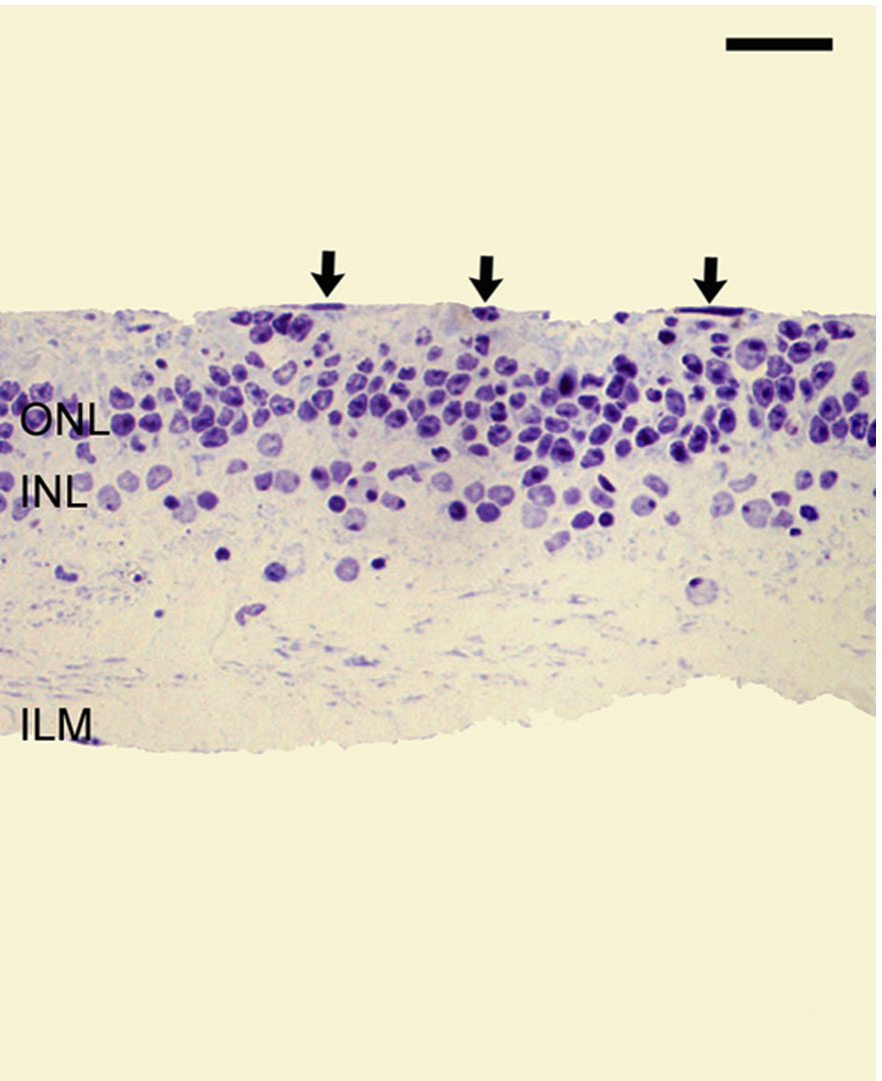
Toluidine blue staining of semithin sections from explants cocultured with the mononuclear fraction. Explants cultured 9 days with the mononuclear fraction (MNF) had more evident cellular disorganization and large multinuclear tissue invasion of the subretinal space (arrows). Scale bar equals 20 µm. Abbreviations: inner nuclear layer (INL); inner limiting membrane (ILM); outer nuclear layer (ONL).

Neuroretinal explants cocultured with MNF did not reveal any noticeable histological changes at 3 and 6 days of culture in comparison with controls. However, all of the explants cultured for 9 days with MNF showed cellular disorganization and larger tissue invasion of the subretinal space accompanied by cellular nuclei (Figure 4).

#### Immunofluorescence microscopy

**Figure 5:**
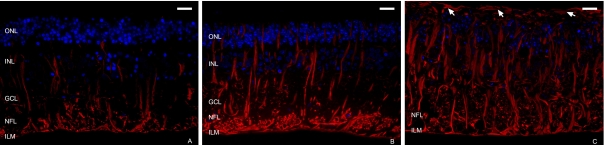
Immunofluorescence staining of semithin sections from explants cocultured with the mononuclear fraction. Antibody against glial fibrillary acidic protein (GFAP; red) and DAPI dye (blue) were used. At 3 (**A**), 6 (**B**), and 9 (**C**) days of culture, the Müller cells were wider and more positive for GFAP than controls. After 9 days in culture (**C**), numerous Müller cell GFAP+ processes invaded the subretinal space, distributed in several fibrous layers (arrows). Scale bar: 20 µm. Abbreviations: GCL is ganglion cell layer; ILM is inner limiting membrane; INL is inner nuclear layer; NFL: nerve fiber layer; ONL is outer nuclear layer.

Immunohistochemical labeling of semithin sections revealed increased GFAP+ reactivity and significant changes in the Müller cells at all culture periods. In the presence of MNF, it was apparent that more Müller cells were wider and were more immunoreactive than the controls (Figures 5A-C). In addition, at culture day 9 (Figure 5C), multiple labeled processes invaded the subretinal space and were distributed in several fibrous layers. There was also an increase in GFAP labeling intensity in the NFL and GCL.

**Figure 6:**
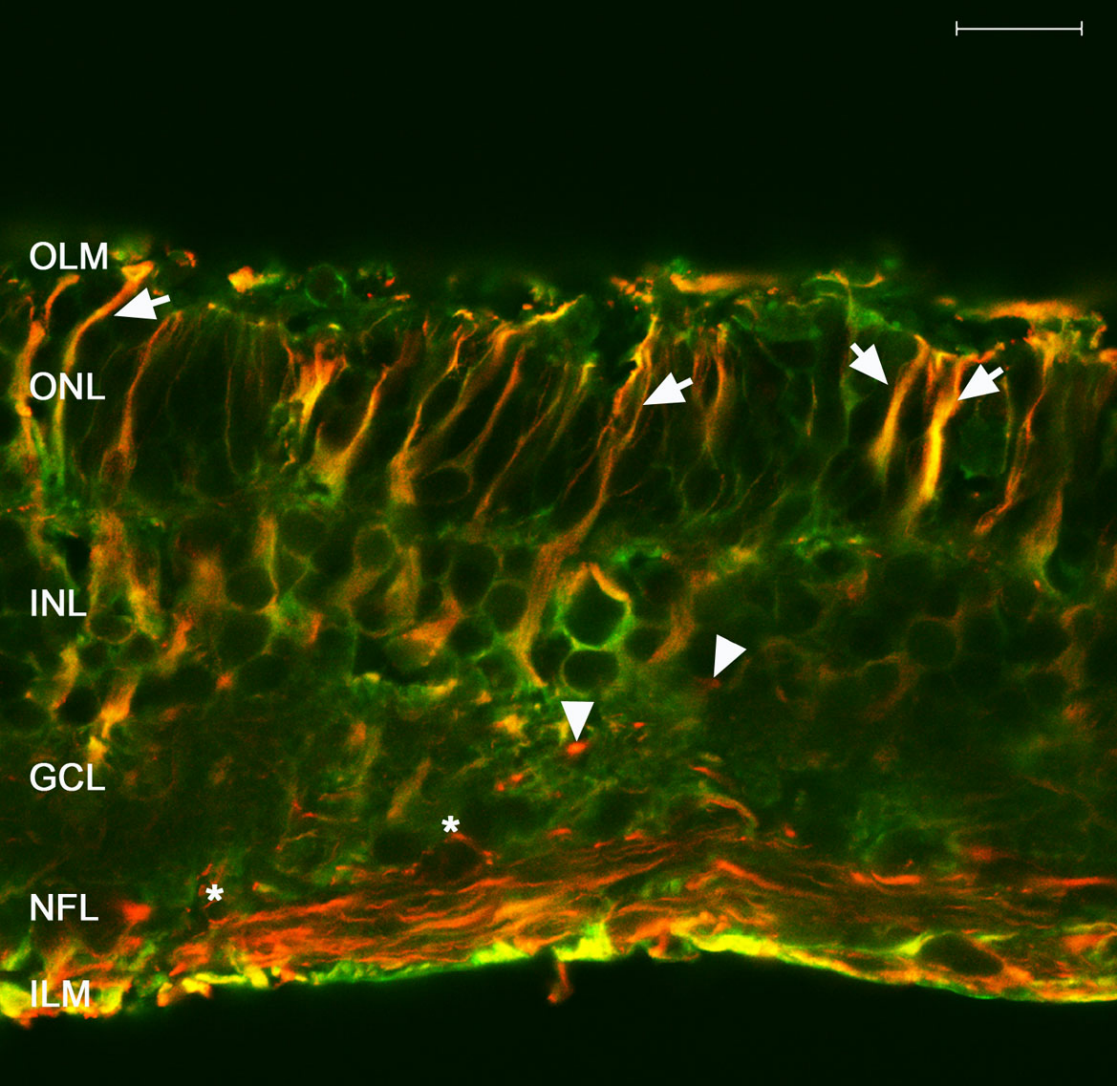
Immunofluorescence staining of cryosections from 6 day cocultured explants. Antibodies against glial fibrillary acidic protein (GFAP; red) and cellular retinaldehyde-binding protein (CRALBP; green) were used. Confocal images revealed an increased expression of GFAP and a decreased CRALBP expression in the neuroretinal external layers. Müller cell processes crossed the outer limiting membrane (OLM; arrows). The cell bodies of GFAP+ astrocytes remained in the nerve fiber layer (NFL; asterisk), but the extensions crossed the neuroretina, reaching the outer layers (arrowheads). Scale bar equals 20 µm. Abbreviations: ganglion cell layer (GCL); inner limiting membrane (ILM); inner nuclear layer (INL); outer nuclear layer (ONL).

Cryosections of explants cocultured with MNF for 6 days were examined by confocal microscopy (Figure 6). Colabeling with both anti-GFAP and anti-CRALBP revealed an increased expression of GFAP and a decreased CRALBP expression in the neuroretinal external layers. Additionally, Müller cell processes crossed the OLM. The cell bodies of GFAP+ astrocytes remained in the NFL, but the extensions crossed the neuroretinal thickness to reach the outer layers.

## Discussion

While the organotypic culture of the neuroretina was originally described to follow cellular and cytoskeletal changes during the culture period [5,27], this model also reproduced some of the cellular changes revealed in experimental RD [2]. Thus, we have used it to analyze the in vitro response of Müller cells to a mononuclear blood fraction as a source of macrophages. The porcine neuroretina was selected because it has many similarities with the human one. These include retinal size, extension, structure, and ultrastructure [28]. Additionally, the holoangiotic vasculature pattern and the distribution of immunocompetent cells [29,30] are quite similar to that of the human eye.

In control cultures, the most important light microscopy findings were early OS degeneration, ganglion cell layer vacuolization, plexiform layer shortening, cellular death, and finally cellular debris and nuclei appearing over the OLM. These changes were similar to the development of subretinal membranes found in RD [2]. Noticeable retinal thinning also occurred during the culture. Similar observations have also been found in other models of RD, both in vivo and in vitro [2,5,31,32]. At 3 and 6 days of culture, OS fragments present in the subretinal space were diminished, similar to that which occurs during longer term RD. In vivo, this is probably the result of phagocytosis by RPE cells and macrophages that invade the subretinal space [31]. The thickening of the ONL can be attributed to regional differences of the explants. Additionally, it could be due to Müller cell gliosis and growth of processes that fill the space left by dying neurons. This gliosis causes a disorganization of the ONL that reduces the packing density of the cells and results in ONL thickening.

Immunofluorescence staining of freshly detached samples was consistent with previous studies [2,27,32,33]. However, another porcine neuroretinal study found that GFAP+ staining was distributed from the NFL to the ONL [5]. We found rapid increases in GFAP expression during the first 3 days of culture, reaching the ONL. These changes were probably associated with photoreceptor cell death that occurs after RD [34]. Subsequently, the expression of the GFAP IF proteins continued to increase in the Müller cell cytoplasm, but the rate of increase diminished. At 6 days, GFAP expression in our cultures was similar to that seen in other studies [2,5]. Finally, cytoplasmic extensions of the Müller cells surrounded photoreceptors IS and invaded the subretinal space, establishing a newly formed tissue at 9 days of culture. This tissue appeared similar to the subretinal membranes developed between 7 and 28 days after experimental RD in cats [2,4] and in porcine neuroretina organotypic culture after 10 days [5]. Previous studies of human specimens [6,8,10] revealed the same increases in GFAP throughout Müller cells.

In explants cocultured with MNF, the glial cell thickness appeared to increase. This needs to be confirmed in quantitative follow-up studies. In addition to the elongation and overgrowth, Müller cells underwent a greater degree of hypertrophy and hyperplasia in the subretinal space when the explants were cocultured with MNF. This response was likely due to interactions of Müller cells with lymphocytes as well as macrophage-like cells [13] that can induce a retinal reactive gliosis. In this model, MNF was the most likely source of the macrophages as these cells are abundant when MNF were cultured alone. Besides Müller cell modifications, astrocytes also underwent some changes, such as growth of extensions that invaded the neuroretinal outer layers by 6 days of culture.

At 9 days of culture, growth of immunoreactive Müller cells processes occupied the space left by dying neurons. At this time, cells seemed to adhere to one another, forming a protective whole that included the rest of surviving neurons. The immunofluorescence and histology images at 9 days of culture with the MNF mimicked the findings obtained in human PVR retinectomy specimens [7,9], showing greater retinal structure disorganization, loss of neuronal nuclei, and GFAP+ reactive gliosis than prior RD models [2,5,31,32].

Previous studies indicated that the breakdown of the blood-ocular barrier and the separation between OS and RPE that occurs in RD are the principal stimuli for the migration of macrophage-like cells into the retina [35-37]. These cells probably originated from blood monocytes [9] and RPE dedifferentiation [36,38]. Furthermore, apoptotic photoreceptors attract macrophages [39], and in vitro studies demonstrated that macrophage-like cells could be activated after interaction with the RPE [17,40]. In the activated state, these inflammatory cells can release multiple proangiogenic and proinflammatory cytokines. One of these cytokines, TNFα, binds to receptors on Müller cells [41] and probably activates them [13]. In addition, TNFα activates microglia and astrocytes in the central nervous system [42].

In summary, we developed an in vitro neuroretina model of Müller cell gliosis that is enhanced by external addition of macrophages. The cellular interactions present in this model could be used for pharmacological assays, testing the efficacy of drugs that can inhibit the retinal scarring process. Cytokines released by activated macrophages will be a target for future studies.
